# Complete Genome Sequences of 18 Paenibacillus larvae Phages from the Western United States

**DOI:** 10.1128/MRA.00966-18

**Published:** 2018-10-04

**Authors:** Bryan D. Merrill, Christopher P. Fajardo, Jared A. Hilton, Ashley M. Payne, Andy T. Ward, Jamison K. Walker, Aziza Dhalai, Cameron Imahara, James Mangohig, Josh Monk, Cristian Pascacio, Padmani Rai, Alicia Salisbury, Kathie Velez, Travis J. Bloomfield, Brett Buhler, Steven G. Duncan, David A. Fuhriman, Josil George, Kiel Graves, Karli Heaton, Hunter L. Hill, Michelle Kim, Bradley K. Knabe, Devin B. Ririe, Spencer L. Rogers, Casey Stamereilers, Michael B. Stephenson, Brittian K. Usher, Colton S. Ward, Jacob M. Withers, Cole K. Wright, Donald P. Breakwell, Julianne H. Grose, Sandra Hope, Philippos K. Tsourkas

**Affiliations:** a Department of Microbiology and Molecular Biology, Brigham Young University, Provo, Utah, USA; b School of Life Sciences, University of Nevada, Las Vegas, Nevada, USA; Georgia Institute of Technology

## Abstract

We present here the complete genomes of 18 phages that infect Paenibacillus larvae, the causative agent of American foulbrood in honeybees. The phages were isolated between 2014 and 2016 as part of an undergraduate phage discovery course at Brigham Young University. The phages were isolated primarily from bee debris and lysogens.

## Abstract (precis)

We present here the complete genomes of 18 phages that infect Paenibacillus larvae, the causative agent of American foulbrood in honeybees. The phages were isolated between 2014 and 2016 as part of an undergraduate phage discovery course at Brigham Young University.

## ANNOUNCEMENT

Paenibacillus larvae is a Gram-positive bacterium that is the causative agent of American foulbrood, the most destructive bacterial disease affecting the honeybee, Apis mellifera (1). As antibiotic-resistant strains are now widespread (2), there is growing interest in phages that infect P. larvae. There are currently 26 complete P. larvae phage genomes in the literature (3–8). Here, we present 18 complete P. larvae phage genomes isolated over the period 2014 to 2016 by students in the Phage Hunters course at Brigham Young University (BYU). Each phage's GenBank accession number, isolation source, geographical provenance, and assembly results are shown in Table 1.

**TABLE 1:** P. larvae phages, GenBank accession numbers, and genome assembly results

Phage name	GenBank accession no.	Isolation source	Location	Genome assembly results		
				Genome length (bp)	DNA-packaging strategy	Avg coverage (×)
Arcticfreeze	MH431932	Bee sample	Idaho	38,518	3′ cos	1,388
Ash	MH454076	Prophage	Provo, UT	56,468	DTR	54
Bloom	MH454077	Bee debris	Spanish Bottom, UT	38,519	3′ cos	101
C7Cdelta	MH431938	Bee sample	Cedar City, UT	55,774	DTR	336
DevRi	MH431933	Bee debris	Spanish Fork, UT	38,520	3′ cos	1,286
Eltigre	MH454078	Bee debris	South Jordan, UT	38,675	3′ cos	1,390
Honeybear	MH431935	Feral bees	Farmington, UT	40,054	3′ cos	77
Genki	MH454082	Bee debris	Orem, UT	38,540	3′ cos	190
Gryphonian	MH431934	Bee debris	Orem, UT	38,541	3′ cos	932
Jacopo	MH454079	Infected hive	Portland, OR	38,526	3′ cos	396
Kawika	MH431936	Bee debris	Provo, UT	40,768	3′ cos	126
Ley	MH454080	Prophage	Provo, UT	56,465	DTR	760
LincolnB	MH454081	Bee debris	Brigham City, UT	40,437	3′ cos	300
Lucielle	MH431937	Dead bee	Idaho	37,947	3′ cos	178
Saudage	MH454083	Bee debris	Lehi, UT	37,962	3′ cos	211
Toothless	MH454084	Bee debris	West Jordan, UT	38,832	3′ cos	240
Wanderer	MH431930	Bee debris	Wisconsin	40,448	3′ cos	1,712
Yerffej	MH431931	Bee debris	Wisconsin	43,126	3′ cos	964

All phages were amplified using P. larvae strain ATCC 9545. Phage DNA was isolated from high-titer lysates using DNA isolation kits (Norgen Biotek, Thorold, ON, Canada). Libraries were prepped with TruSeq Nano DNA HT sample preparation kits (Illumina, Inc., Hayward, CA, USA) and then run on a single lane in parallel and barcoded. Genomes were sequenced in the BYU DNA Sequencing Center using the Illumina HiSeq 2500 platform with 250-bp paired-end reads and assembled using Geneious 8 (Biomatters, Inc., Auckland, New Zealand) with medium-low sensitivity/fast and checking for contig circularization. Only genomes that produced circularized contigs were considered complete and published. Genomes were manually annotated by students at the University of Nevada Las Vegas (UNLV) with DNA Master, as previously described (9).

Scanning electron micrographs show that all 18 phages are members of the family Siphoviridae. All the genomes are linear double-stranded DNA molecules. Phages Ash, C7Cdelta, and Ley use the direct terminal repeat (DTR) DNA-packaging strategy, while the other 15 phages use the “cohesive ends with 3′ overhangs” (cos) DNA-packaging strategy (10, 11). The 3′ overhangs were identified by sequence similarity with previously published phages (3–8). The overhangs are CGACTGCCC for Arcticfreeze, Bloom, DevRi, Eltigre, Genki, Gryphonian, Honeybear, Jacopo, Kawika, Lucielle, Saudage, and Toothless and CGACGGCCC for LincolnB and Wanderer. The genome ends of Yerffej are still under investigation. For the DTR phages, the DTR sequence was visually identified using Pile-up Analysis Using Starts & Ends (PAUSE) (http://cpt.tamu.edu/computer-resources/pause) and Geneious by looking for a sharply delimited region with double coverage depth (11).

Genome length is bimodal, with the cohesive end phages having genomes in the 37- to 43-kb range and the DTR phages having genomes in the 55- to 56-kb range, which is consistent with previously published P. larvae phages (3–8). All phages encode a large terminase, a portal protein, a major capsid protein, two tail assembly proteins, a tail tape measure protein, several tail proteins, and an *N*-acetylmuramoyl-l-alanine amidase. The tail assembly proteins appear to have a predicted translational frameshift similar to that of the G and G-T genes in phage lambda (12, 13) located in the 3′ region of the upstream tail assembly protein (gp12 in the phages with cohesive ends, gp14 in the DTR phages). We tentatively identify the heptanucleotide slippery sequence as AAAAAAA in Arcticfreeze, Bloom, DevRi, Eltigre, Genki, Gryphonian, Honeybear, Jacopo, Kawika, Lucielle, Saudage, Toothless, and Yerffej, GGAAAAA in LincolnB and Wanderer, and TAAAAAA in Ash, C7Cdelta, and Ley.

### Data availability.

GenBank accession numbers are listed in Table 1.
